# Supplementary material for: Interaction between the PNPLA3 Gene and Nutritional Factors on NAFLD Development: The Korean Genome and Epidemiology Study
Source: Nutrients. 2022 Dec 28;15(1):152. doi: 10.3390/nu15010152 (PMC9824262; doi:10.3390/nu15010152)
Supplement: Supplementary file 1 [file nutrients-15-00152-s001.zip › nutrients-2091779-supplementary.pdf]

Supplementary Appendix for

Interaction between PNPLA3 gene and Nutritional factors on NAFLD development: the Korean Genome and Epidemiology Study (KoGES)

Coauthored by Sooyeon Oh, Jooho Lee*, Sukyung Chun, Ja-Eun Choi, Mi Na Kim, Young Eun Chon, Yeonjung Ha, Seong-Gyu Hwang, Sang-Woon Choi and Kyung-Won Hong*

***Correspondence:**

Jooho Lee, M.D., Ph. D. (E-mail: ljh0505@cha.ac.kr),

Kyung-Won Hong, Ph. D. (Email: kyungwon.hong@theragenbio.com)

Supplementary Table S1. Recommended daily intake of each nutrient according to the 2020 Korean Dietary Reference Intakes

Nutrients	Median value
**Energy**	2000 kcal
**Carbohydrate**	250 g
**Protein**	55 g
**Fat**	25 g
**Sodium**	2000 mg
**Potassium**	3500 mg
**Calcium**	700 mg
**Phosphorus**	700 mg
**Zinc**	8 ug
**Iron**	15 mg
**Vit.A**	700 R.E
**Carotene**	1800 ug
**Vit.B1**	1.4 mg
**Vit.B2**	1.4 mg
**Niacin**	15 mg
**Vit.B6**	1.4 mg
**Folate**	400 ug
**Vit.C**	100 mg
**Vit.E**	12 mg
**Ash**	20 mg
**Cholesterol**	300 mg

Ministry of Health & Welfare's research project, 2020 Korean Dietary Reference Intakes, http://www.kns.or.kr/News/Notice_view.asp?mode=mod&restring=%252FNews%252FNotice.asp%253Fxsearch%253D0%253D%253Dxrow%253D10%253D%253Dpage%253D1&idx=864&page=1&xsearch=1&cn_search=

Supplementary Table S2. Genetic risk of *the PNPLA3* rs738409 *G* allele for non-alcoholic fatty liver disease (NAFLD) occurrence in genome-wide association studies (GWAS) analyses

	Additive model		Dominant model		Recessive model	
	**OR (95% CI)**	***P* - value**	**OR (95% CI)**	***P* - value**	**OR (95% CI)**	***P* - value**
***PNPLA3* rs738409 allele**						
C allele	**1**		**1**		**1**	
G allele	**1.22 (1.15~1.30)**	**1.96×10^{-10}**	**1.27 (1.15~1.39)**	**1.24×10^{-6}**	**1.36 (1.22~1.52)**	**1.82×10^{-8}**

Abbreviations: NAFLD, non-alcoholic fatty liver disease; 95% CI, 95% confidence interval; OR, odds ratio; GWAS, Genome-wide association studies

; *PNPLA3*, patatin-like phospholipase domain-containing 3

Supplementary Table S3. Proportion of patients with *high intake of each nutrient

	Total population (n=15,725)	NALFD (n=2923)	Control (n=12802)	*p*-value
Energy	29.31	26.89	29.86	0.002
Carbohydrate	79.28	80.53	79.00	0.067
Protein	53.21	47.76	54.45	<0.001
Fat	54.07	44.27	56.31	<0.001
Sodium	59.84	57.41	60.4	0.003
Potassium	9.96	9.75	10.01	0.7
Calcium	11.83	12.08	11.78	0.67
Phosphorus	71.88	69.14	72.5	<0.001
Zinc	42.38	40.71	42.77	0.04
Iron	11.46	11.7	11.40	0.67
Vitamin A	16.87	16.87	16.87	1
Carotene	54.52	52.93	54.88	0.057
Vitamin B1	15.75	13.1	16.36	<0.001
Vitamin B2	11.4	10.33	11.65	0.047
Niacin	41.69	35.99	42.99	<0.001
Vitamin B6	56.58	53.13	57.37	<0.001
Folate	6.54	7.15	6.41	0.15
Vitamin C	43.56	43.28	43.63	0.74
Vitamin E	14.4	13.51	14.61	0.13
Ash	16.93	16.8	16.96	0.86
Cholesterol	13.14	12.04	13.4	0.054

Values are presented as percent (%).

*For each nutrient, a daily intake above the recommended daily value was defined as high intake and below as low intake. The recommended daily values used in this study are presented in Supplementary Table S1.

Supplementary Table S4. non-alcoholic fatty liver disease (NAFLD) case frequencies by nutrient consumption level in the *PNPLA3 risk group (rs738409 GG + GC) (n = 10,530) determined by univariate analyses

Nutrient	High intake**			Low intake**			OR	*P-value*
	NAFLD (n)	Control (n)	Proportion of NAFLD (%)	NAFLD (n)	Control (n)	Proportion of NAFLD (%)		
Energy(kcal)	556	2532	18.01%	1536	5906	20.64%	0.946	0.381
Protein(g)	982	4579	17.66%	1110	3859	22.34%	0.821	0.001
Fat(g)	913	4746	16.13%	1179	3692	24.20%	0.755	<0.0001
Carbohydrate(g)	1689	6634	20.29%	403	1804	18.26%	1.037	0.617
Ca(mg)	245	965	20.25%	1847	7473	19.82%	0.997	0.972
P(mg)	1418	6084	18.90%	674	2354	22.26%	0.851	0.009
Fe(mg)	232	962	19.43%	1860	7476	19.92%	0.98	0.817
K(mg)	202	817	19.82%	1890	7621	19.87%	0.991	0.922
Vit.A (R.E.)	338	1416	19.27%	1754	7022	19.99%	0.905	0.19
Sodium (mg)	1161	5119	18.49%	931	3319	21.91%	0.771	<0.0001
Vit.B1(mg)	264	1372	16.14%	1828	7066	20.55%	0.881	0.122
Vit.B2(mg)	214	962	18.20%	1878	7476	20.08%	0.959	0.649
Niacin(mg)	740	3630	16.93%	1352	4808	21.95%	0.8	<0.0001
Vit.C(mg)	907	3684	19.76%	1185	4754	19.95%	0.97	0.594
Zinc(ug)	844	3591	19.03%	1248	4847	20.48%	0.948	0.354
Vit.B6(mg)	1101	4842	18.53%	991	3596	21.60%	0.823	0.001
Folate(ug)	135	527	20.39%	1957	7911	19.83%	0.961	0.73
Carotene(ug)	1092	4622	19.11%	1000	3816	20.76%	0.878	0.022
Ash(mg)	335	1421	19.08%	1757	7017	20.03%	0.833	0.017
Vit.E(mg)	278	1213	18.65%	1814	7225	20.07%	1.016	0.849
Cholesterol(mg)	241	1110	17.84%	1851	7328	20.17%	1.012	0.892

Abbreviations: NAFLD, non-alcoholic fatty liver disease; OR, odds ratio

*In the *PNPLA3* risk group, The proportion of NAFLD patients was 19.87% (2,092/10,530).

** For each nutrient, daily intake above the recommended daily value was defined as high intake and below that as low intake. The recommended daily values used in this study are presented in Supplementary Table S1.

Supplementary Table S5. Non-alcoholic fatty liver disease (NAFLD) case frequencies by nutrient consumption level in the *PNPLA3 non-risk group (rs738409 CC) (n = 5,195) determined by univariate analyses

Nutrient	High intake**			Low intake**			OR	*P-value*
	NAFLD (n)	Control (n)	Proportion of NAFLD (%)	NAFLD (n)	Control (n)	Proportion of NAFLD (%)		
Energy(kcal)	230	1291	15.12%	601	3073	16.36%	0.943	0.551
Protein(g)	414	2392	14.75%	417	1972	17.46%	0.863	0.094
Fat(g)	381	2463	13.40%	450	1901	19.14%	0.794	0.009
Carbohydrate(g)	665	3479	16.05%	166	885	15.79%	0.94	0.577
Ca(mg)	108	543	16.59%	723	3821	15.91%	0.954	0.715
P(mg)	603	3198	15.86%	228	1166	16.36%	0.971	0.77
Fe(mg)	83	464	15.17%	748	3900	16.09%	1.226	0.115
K(mg)	110	498	18.09%	721	3866	15.72%	0.901	0.466
Vit.A (R.E.)	155	744	17.24%	676	3620	15.74%	1.075	0.517
Sodium (mg)	517	2613	16.52%	314	1751	15.21%	1.017	0.851
Vit.B1(mg)	119	722	14.15%	712	3642	16.35%	0.889	0.339
Vit.B2(mg)	88	529	14.26%	743	3835	16.23%	0.819	0.15
Niacin(mg)	312	1874	14.27%	519	2490	17.25%	0.843	0.057
Vit.C(mg)	358	1901	15.85%	473	2463	16.11%	1.016	0.859
Zinc(ug)	346	1884	15.52%	485	2480	16.36%	0.917	0.331
Vit.B6(mg)	452	2502	15.30%	379	1862	16.91%	0.865	0.099
Folate(ug)	74	293	20.16%	757	4071	15.68%	1.325	0.074
Carotene(ug)	455	2404	15.91%	376	1960	16.10%	0.976	0.783
Ash(mg)	156	750	17.22%	675	3614	15.74%	1.032	0.782
Vit.E(mg)	117	657	15.12%	714	3707	16.15%	0.985	0.9
Cholesterol(mg)	111	605	15.50%	720	3759	16.08%	1.005	0.97

Abbreviations: NAFLD, non-alcoholic fatty liver disease; OR, odds ratio

*In the *PNPLA3* non-risk group, the proportion of NAFLD patients was 15.99% (831/5,195).

** For each nutrient, daily intake above the recommended daily value was defined as high intake and below that as low intake. The recommended daily values used in this study are presented in Supplementary Table S1.
